# Still finding ways to augment the existing management of acute and chronic kidney diseases with targeted gene and cell therapies: Opportunities and hurdles

**DOI:** 10.3389/fmed.2023.1143028

**Published:** 2023-03-07

**Authors:** Peter R. Corridon

**Affiliations:** ^1^Department of Immunology and Physiology, College of Medicine and Health Sciences, Khalifa University, Abu Dhabi, United Arab Emirates; ^2^Biomedical Engineering, Healthcare Engineering Innovation Center, Khalifa University, Abu Dhabi, United Arab Emirates; ^3^Center for Biotechnology, Khalifa University, Abu Dhabi, United Arab Emirates

**Keywords:** acute kidney disease, chronic kidney disease, gene therapy, cell therapy, renal

## Abstract

The rising global incidence of acute and chronic kidney diseases has increased the demand for renal replacement therapy. This issue, compounded with the limited availability of viable kidneys for transplantation, has propelled the search for alternative strategies to address the growing health and economic burdens associated with these conditions. In the search for such alternatives, significant efforts have been devised to augment the current and primarily supportive management of renal injury with novel regenerative strategies. For example, gene- and cell-based approaches that utilize recombinant peptides/proteins, gene, cell, organoid, and RNAi technologies have shown promising outcomes primarily in experimental models. Supporting research has also been conducted to improve our understanding of the critical aspects that facilitate the development of efficient gene- and cell-based techniques that the complex structure of the kidney has traditionally limited. This manuscript is intended to communicate efforts that have driven the development of such therapies by identifying the vectors and delivery routes needed to drive exogenous transgene incorporation that may support the treatment of acute and chronic kidney diseases.

## 1. Introduction

Renal dysfunction can be acute, chronic, or end-stage, manifesting in several forms. The most prevalent cases arise from congenital disorders (1, 2); nephrotoxicity (3); ischemia–reperfusion injury (4, 5); systolic hypotension and hemorrhage (6); hypertension (7); trauma (8); essential mineral deficiencies (9); malignancies (10); diabetes (11, 12); and viral infections, as observed with the COVID-19 pandemic (13, 14). Paradoxically, hospitalization and the complex relationship between various forms of kidney injuries are additional factors that can contribute to renal dysfunction. For decades, clinicians have been aware of the risk of patients, with and without underlying kidney injury, developing hospital-acquired kidney malfunction (15). They have also been aware of the complex connection between acute kidney injury (AKI) and chronic kidney disease (CKD), whereby they are closely linked and likely promote one another. For instance, CKD is a reputed risk factor for developing AKI during hospitalization, while there is a growing body of evidence illustrating how AKI accelerates the progression of CKD in critically ill patients (16), particularly hospitalized COVID-19 patients (17).

From a global perspective, it is estimated that AKI affects approximately 13 million people annually, contributing to nearly 1.7 million annual deaths (18). Traditionally, AKI is a critical stage in injury progression because of its reversibility (19). In comparison, CKD affects over one-tenth of the general population worldwide (20), and eventually, these conditions contribute to 5–8 million patients with end-stage renal disease (ESRD) requiring renal replacement therapy (21). AKI is a critical stage in injury progression because of its reversibility (21). Beyond this stage, treatment options are limited to renal replacement therapy, as the dysfunction has progressed to either CKD or, unfortunately, ESRD. It was previously thought that AKI, a sudden reduction in renal function, was fully reversible in all patients (22). Nevertheless, recent research has gone against this notion based on studies conducted on individuals with reduced filtration capacities who are more prone to ESRD progression and mortality than a reversal of the condition (23, 24).

These facts highlight significant clinical problems that arise from acute and chronic disorders. Furthermore, from a financial perspective, these patients often require long-term hospitalization, which imposes substantial burdens on the healthcare systems related to the etiologies of these disorders and their complex and debilitating interconnected nature. Likewise, these conditions lead to enhanced levels of morbidity and reductions in quality of life. Overall, morbidity and mortality are expected to rise exponentially with the growing rates of diabetes and cardiovascular diseases. Given that current treatments are mainly preventive strategies and early detection and intervention can be difficult in asymptomatic patients with these conditions, there is a definite need for alternative strategies to address the growing prevalence and subtle progression of renal dysfunction and ultimately reduce the need for renal replacement therapy (5, 25–28).

In the search for such strategies, significant efforts are being devised to augment the present-day management of kidney disease using novel regenerative strategies. For example, gene- and cell-based approaches that utilize recombinant peptides/proteins, gene, cell, organoid, and RNAi technologies have shown promising outcomes primarily in experimental models (25). Accompanying efforts have also been devised to facilitate the development of efficient gene- and cell-based techniques. This article is intended to convey efforts that have advanced these alternative forms of therapy by highlighting vectorization and mechanisms that can elicit genetic modifications that may support the treatment of acute and chronic kidney diseases.

## 2. Efforts to devise effective genetic alterations in the kidney

### 2.1. Recombinant peptides and proteins

Various methods have been proposed to deliver exogenous genes to mammalian cells. For the kidney, attempts have been made to protect and repair renal function by targeting single genetic loci with purified protein products, plasmids, recombinant growth factors, and viruses encoding peptides and proteins. Intravenous doses of human growth factor (HGF), which has anti-fibrotic properties, have promoted kidney repair in rodents with CKD (29, 30). Injections of IL-18BP, a recombinant interleukin, improved renal function, restored tubular morphology, and decreased tubular necrosis and apoptosis in small animal models (31). Cell-based approaches conducted with intrarenal injections of human placenta-derived stem cells have also ameliorated damage in ischemia–reperfusion settings of AKI (32).

Single intravenous doses of plasmids encoding human growth factor (HGF) have also been shown to improve tissue regeneration and protect tubular epithelial cells from injury and apoptosis during acute renal failure (33). In such earlier studies, HGF also helped preserve renal structure in chronic injury models by activating matrix degradation and reducing fibrosis (34–36). Researchers have tested growth hormone-releasing hormone (GHRH) plasmid-based therapy in feline and canine chronic injury models. GHRH-treated animals displayed better levels of erythropoiesis, urea and creatinine clearances compared to controls (37), as well as more recent findings related to its therapeutic effect in CKD patients (38).

It has been well-established that adenovirus and adeno-associated virus vectors are two of the most efficient systems for transducing non-dividing cells (39) and have been used to target a variety of genetic loci. Other experimental studies have used adeno-based vectors for gene transfer. Lately, such vectors have displayed the long noncoding RNA-H19-derived attenuation of acute ischemic kidney injury (40) and the mediation of AKI to CKD progression (41). These vectors have also helped preserve renal microvascular morphology and suppress the progression of AKI *via* the upregulation of vascular endothelial growth factor (VEGF) and angiopoietin (42). Interestingly, the inhibition of VEGF also promoted structural and functional improvements in diabetes-induced chronic kidney disease (43, 44). These findings support the long-derived notion that repairing ischemic and toxic renal injuries depended critically on regulating a redundant, interactive network of cytokines and growth factors (45). Thus, it would be of value to devise a system that could reliably modulate gene expression levels to return kidney function to near-normal baseline levels without inducing viral-derived toxicity. However, despite its benefits regarding kidney function recovery, recombinant agents have short half-lives and require large doses (46). Further studies are needed to demonstrate consistent safety and effectiveness levels before these experimental techniques become clinical practice (47).

### 2.2. Cell and organoid transplantation

Cell therapy is another option to improve tissue/organ regeneration. Research efforts initially focused on cell transfer for bone marrow and organ transplantation, blood transfusion, and *in vitro* fertilization (48). Nowadays, this technique is being developed to facilitate the repair/replacement of damaged and lost compartments in solid organs. This regenerative strategy transplants cells, which deliver genes of interest, to targeted organs. To achieve this purpose, investigators use the following cells: stem or progenitor cells; mature, functional cells from humans or animals; and genetically modified and transdifferentiated cells (48–51). More recently, organoids, transdifferentiated three-dimensional cell clusters, arose as another promising option to enhance or restore kidney function (52–54).

Papazova et al. published a meta-analysis of CKD and cell therapies (55). This analysis demonstrated that more than half of all cell-based studies focused on the therapeutic effects of single intravenous doses of mesenchymal stem cells. About a third of the studies investigated the preventive benefits of such therapies, while half of the studies focused on their therapeutic benefits. For instance, in AKI animal models, mesenchymal stem cells improved renal function (56–58). Even though the specific mechanisms of action are still under investigation, these cells helped reduce renal fibrosis, improve remodeling, and promote neoangiogenesis (59). Kelly et al. also helped restore renal function using undifferentiated reprogrammed cells to generate sera amyloid A proteins in ischemia–reperfusion, plus gentamicin- and cisplatin-based nephrotoxicity acute injury rat models (60).

Additional efforts have also reported the successful differentiation of embryonic and induced pluripotent stem cells into tubular, glomerular, and whole nephron organoids (61–68). A greater understanding of the roles of key signaling pathways has also allowed investigators to differentiate stem cell niches into various lineages. We believe that shortly, organoids derived from patients’ cells will be able to repopulate decellularized renal scaffolds and printed tissues or even be injected back into the patients to restore their native dysfunction (69–71). Nevertheless, many technical (72–78) and ethical (79–87) issues still need to be solved in this field. It is well-established that embryonic stem cell technology offers hope for new therapies, yet societal and moral incongruences limit their use. Teratoma, a hallmark of pluripotency (89–91), is a significant concern after implantation. The ability to culture and manipulate human stem cells indefinitely while simultaneously governing their differentiation characteristics offers excellent possibilities for the future of medicine (92–94).

### 2.3. RNA interference therapy

Another option within the growing arsenal of gene and cell therapy applications is RNA interference (RNAi). The discovery of mammalian RNAi is one of the most promising therapeutic strategies because it enables the silencing of any gene (95). RNAi is an advantageous technique, as it is easier to silence deficient and non-functional genes than replace them (96). Moreover, RNAi is the most practical approach thus far, as it is relatively low cost, highly specific, and can inhibit multiple genes of various pathways simultaneously (97). This technology can help identify complex genetic loci essential to human pathology.

RNAi is an endogenous process that allows cells to regulate their genetic activity. This process remains central to gene expression and the defense against mutagenesis generated from viral genes and transposons (98). The primary methods that induce exogenous RNAi-based gene silencing utilize micro-RNA (miRNA), small interfering RNA (siRNA), and small hairpin RNA (shRNA) systems. Since Napoli and Jorgensen first reported on this phenomenon in 1990 (99), there has been a growing interest in using RNAi technology to improve renal health (95). This interest has directed RNAi-based research focused on improving the study and management of kidney disease by identifying miRNA targets and AKI biomarkers. It has also prompted interest in improving the delivery of exogenous silencing mediators and siRNA and shRNA targets to either reduce or protect against renal injury. Currently, lipid nanoparticles are the most frequently used formulation to mediate silencing (100), and further work has been proposed to determine *in vivo* silencing efficiencies and investigate other small RNAs that can affect post-transcriptional gene silencing (101, 102).

From a diagnostic standpoint, several studies have provided fundamental insight into renal injury biomarkers. Valadi et al. showed that miRNAs recovered from urinary exosomes provide information about the kidney in standard and injury settings (103). Zhou et al. showed that miR-27b and miR-192 in these urinary vesicles could differentiate between glomerular and tubular damage (104). Also, from a therapeutic standpoint, exosomes containing miRNAs can enter recipient cells by membrane surface proteins. This phenomenon offers a new mechanism for cell–cell communication and gene delivery (105–111). In a study by Cantaluppi et al., microvesicles enriched with pro-angiogenic miR-126 and miR-296 were injected into the vein, enhanced tubular cell proliferation, and reduced apoptosis and leukocyte infiltration (112). In AKI settings, such silencing has demonstrated that the caspase-3 siRNA improved ischemic reperfusion (IR) injury with reduced caspase-3 expression and apoptosis, better renal oxygenation and acid–base homeostasis, and the silencing IKKβ using siRNA diminished inflammation and protected the kidneys against IR injury (113). Whereas, in a glomerulonephritic chronic injury model, MAPK1 suppression remarkably improved kidney function, reduced proteinuria, and ameliorated glomerular sclerosis (113).

RNAi therapy could be a valuable surrogate for treating patients with AKI by reducing the uptake of nephrotoxins, ameliorating immunologic response mechanisms, and downregulating harmful disease mediators (114–116). Such characteristics have prompted interest in the knockdown of dynamin-2 (Dyn2) and low-density lipoprotein-related protein 2 (LRP2). Dyn2 is a critical component of the endocytic pathway (117–119), and its knockdown blocks clathrin-coat-dependent endocytosis and coat-independent fluid phase probe uptake in several epithelial cell lines (120). In animal models, silencing LRP2 reduced gentamicin toxicity in proximal tubule epithelial cells (121–123). In a rat model of kidney transplantation, caudal vein administration of siRNAs, which targeted connective tissue growth factor (CTGF), decreased renal fibrosis (124). CTGF is an essential pro-fibrotic cofactor that is downstream from TGF-β. Electroporation also enhanced the delivery of siRNA targeted to TGF-β1, significantly reducing glomerular matrix deposition and proteinuria four and 6 weeks after anti-Thy-1 administration (124, 125).

In other studies, which have investigated the renotherapeutic potential of siRNA technology (126), siRNA sequences were systemically delivered to inhibit the expression of p53. This strategy significantly reduced ischemia-induced p53 upregulation and helped attenuate ischemic and cisplatin-induced AKI (127, 128). The oligonucleotides used to facilitate RNAi contained stabilizing modifications with a relatively low affinity for albumin and other plasma proteins. Such modifications diminished their hepatic distribution and degradation in serum and facilitated their renal clearance and endocytic tubular uptake (128). This fact limits the class of therapeutic siRNAs for such procedures because of the natural tendency of systemically delivered materials to accumulate within the liver.

In comparison, the expression of transgenic shRNA targeting the proapoptotic BIM gene prevented the development of polycystic kidney disease in BCl-2 deficient mice (129). However, the mortality rate in this study was high. Additional research is required to identify whether the high mortality rate was due to the sequence of the shRNA.

## 3. Mechanisms for exogenous transgene expression in mammalian cells

One major challenge to developing gene- and cell-based strategies is our need to understand their mechanisms of action. Regardless of the performance of recombinant peptides, DNA vectors, stem cells, and RNAi agents, mechanisms related to each approach still need to be uncovered (47, 69, 130–135). This gap in knowledge makes it difficult to optimize these techniques. Nevertheless, the basic principles for successful transgene expression have been documented (130–134, 136–142). All such therapies rely on efficiently delivering exogenous genes to widespread cellular targets. The techniques discussed earlier have achieved this by directly using DNA/RNA strands or inserting these molecules into gene transport vehicles. Once the genetic materials enter the nuclei, they either aid or inhibit the expression of the gene product(s) of interest in transformed cells and their progeny.

Likewise, the overall efficacy of RNAi in inducing gene silencing in any cell depends on the ability of the dsRNA reagent to access the subcellular compartment containing the RNA-induced silencing complex (RISC) and other components of the RNAi machinery (143, 144). This subcellular compartment is in the perinuclear region of the cytoplasm. However, if cell transplantation mediates transgene expression, the gene delivery process will rely on integrating the delivered cells, native cellular division, and intercellular communication. Furthermore, the goal is to facilitate gene expression/inhibition once exogenous cells are integrated into tissues and organs (145, 146).

For instance, previous work suggests that the effectiveness of gene therapies using adenoviral (147) and siRNA (148) vectors depends on the dose and timing of transgene administration. Such dependence drives variations in drug concentrations at the respective sites of the gene expression and silencing machinery.

It is, therefore, essential to understanding how effective concentrations within the cytoplasm affect therapeutic potency based on dosing and timing of transgene administrations. This factor is a topic of practical importance, as the mechanism(s) will determine the intracellular fate of exogenous transgenes from non-viral, viral, and cellular sources and aid the development of effectual medical strategies that can control the duration and extent of induced genetic traits. Alternatively, for approaches that focus on whole organ engineering and re-engineering, additional insights are needed into the mechanisms behind the successful repopulation of tissue and organ templates (65). Researchers must also determine the characteristics required to facilitate exogenous genetic and cellular harmony for viable transplantable kidneys before these findings can translate into clinical practice.

## 4. Key aspects to facilitate advancements in renal genetic medicine

### 4.1. The development of efficient delivery techniques

Over the past 30 years, many methods have been proposed to deliver exogenous genes and cells to target organs (32, 39, 46, 97, 100, 102, 130, 142, 149–157). From a fundamental viewpoint, these techniques seek to provide inexpensive and rapid alternatives to pronuclear microinjection-derived transgenic models and platforms for translational studies (121). However, a limiting step in this process is the need for more reliable delivery systems. Several reports have indicated inconsistent outcomes regarding the effectiveness of existing gene and cell transfer techniques. Studies in the kidney have illustrated this variability (155, 156, 158–164). In general, an *in vivo* gene and cell transfer system’s success relies on various factors. The factors include:

- the ability to deliver vectors to the target cells/organ;
- the time the target cell/organs take to express the exogenous materials; and
- the number of cells/organs that express the required phenotype.

Other essential factors are the resulting expression levels, cellular turnover rates, the reproducibility of the process, and the severity of the injury that may result from it (95, 130). Thus, most existing strategies remain experimental (165–168).

Researchers must consider organ morphology and function variations as crucial elements to improve the overall efficacy of delivery strategies (169, 170). Thus, efficient gene and cellular therapies for treating kidney diseases remain challenging (47, 171–175). The structure of the renal vasculature and its unique characteristics are prominent limiting factors. Systems focusing on proximal tubular epithelial cellular uptake could be helpful (175–177). However, a potential drawback to this technique is the variations in the glomerular permeability of different molecules (178–183). Likewise, the unknown degree to which these cells are accessible for gene delivery at the basolateral surface *via* the peritubular capillaries provides another level of complication. Studies using adenovirus vectors have demonstrated the need to improve our understanding of renal physiology and our ability to manipulate it.

Intra-arterial kidney injections, pre-chilled for extended periods, facilitated transgene expression within the cortical vasculature (184). Combining the pre-chilling treatment with vasodilators provided gene transfer in the outer medulla’s inner and outer strips (184). Other studies have successfully presented adenoviral vector delivery to rat glomerular and tubular compartments by infusions into the right renal artery (185, 186). This technique provided high levels of transgene expression for 2–4 weeks without causing significant damage (187, 188). Analogous concentrations of the same adenovirus vector were suspended in different volumes and delivered to the kidney *via* arterial injections and pelvic catheter infusions. This approach facilitated transgene expression in distinct kidney regions (188, 189). After injecting vectors into the aorta at a location proximal to the left renal artery, the investigators observed transgene expression only in proximal tubular cells.

Tail vein and retrograde ureteral adenovirus infusions that target aquaporin water channels also reported different expression levels, which depended on the transgene infusion site (130, 156). Aquaporin 1 transgenes were expressed in apical and basolateral membranes of proximal tubule epithelial cells in the renal cortex but not in the glomerulus, loop of Henle, or collecting duct. Conversely, ureteral and renal papilla transgene expression was reported through ureteral infusions. The researchers also reported less intense and patchy expression in cortical collecting ducts. Ashworth et al. (190) and Tanner et al. (161) explored the direct transfer of adenovirus vectors that carried transgenes into individual nephron segments using micropuncture techniques. They observed site-specific transgene expression within the injected tubules or vascular welling points. These results also demonstrated the utility of intravital fluorescent multiphoton microscopy to monitor protein expression in live animals directly. However, one limitation of the approach was that the injection sites were the only places where the investigators found transgene expression.

These studies further highlight the challenge of introducing genes into multiple renal cell types due to the intricate anatomy of the kidney, even when using the same type of vector. Results depend on the transgene infusion site, volume, and rate, as well as the organ temperature and the use of vasodilators. Hydroporation may address these challenges by increasing vascular permeability and thus efficiently delivering exogenous substances throughout the kidney. Hydrodynamic fluid delivery impacts fluid pressures within thin, stretchable capillaries (191, 192). The enhanced fluid flow generated from pressurized injections produces rapid and high fluctuations in blood circulation. Theoretically, it increases the permeability of the capillary endothelium and epithelial junctions by generating transient pores in plasma membranes that facilitate the cellular internalization of macromolecules of interest (47, 191, 193). The unique anatomy of the kidney provides various innate delivery pathways (artery, vein, and ureter) that may be ideal for hydrodynamic gene delivery. In our recent reports, this delivery method provided efficient and lengthy plasmid and viral expression in live rat kidneys (130, 142, 194) and facilitated protection against moderate forms of ischemia–reperfusion injury (154, 195–197). A summary of delivery methods and associated vectors is presented in Table 1.

**Table 1:** An overview of delivery methods and associated vectors.

Infusion Site	Infusion Method	Infusion Compound	Auxiliary Gene Enhancer
Tail vein	Systemic injection (normal volume and pressure)	Plasmid and viral vectors, and cells	None reported
Renal artery, renal vein, renal pelvis, and ureter	Low pressure injections Hydrodynamic injections	DNA particles, liposomes, polycations, stem cells, and viral vectors	Electroporation, microbubble cavitation, ultrasound cavitation, ultrasound and microbubble coupled cavitation
Renal capsule	Micropuncture and blunt needle injections	Viral vectors	None reported

### 4.2. Exogenous transgene vectors

**Figure 1:**
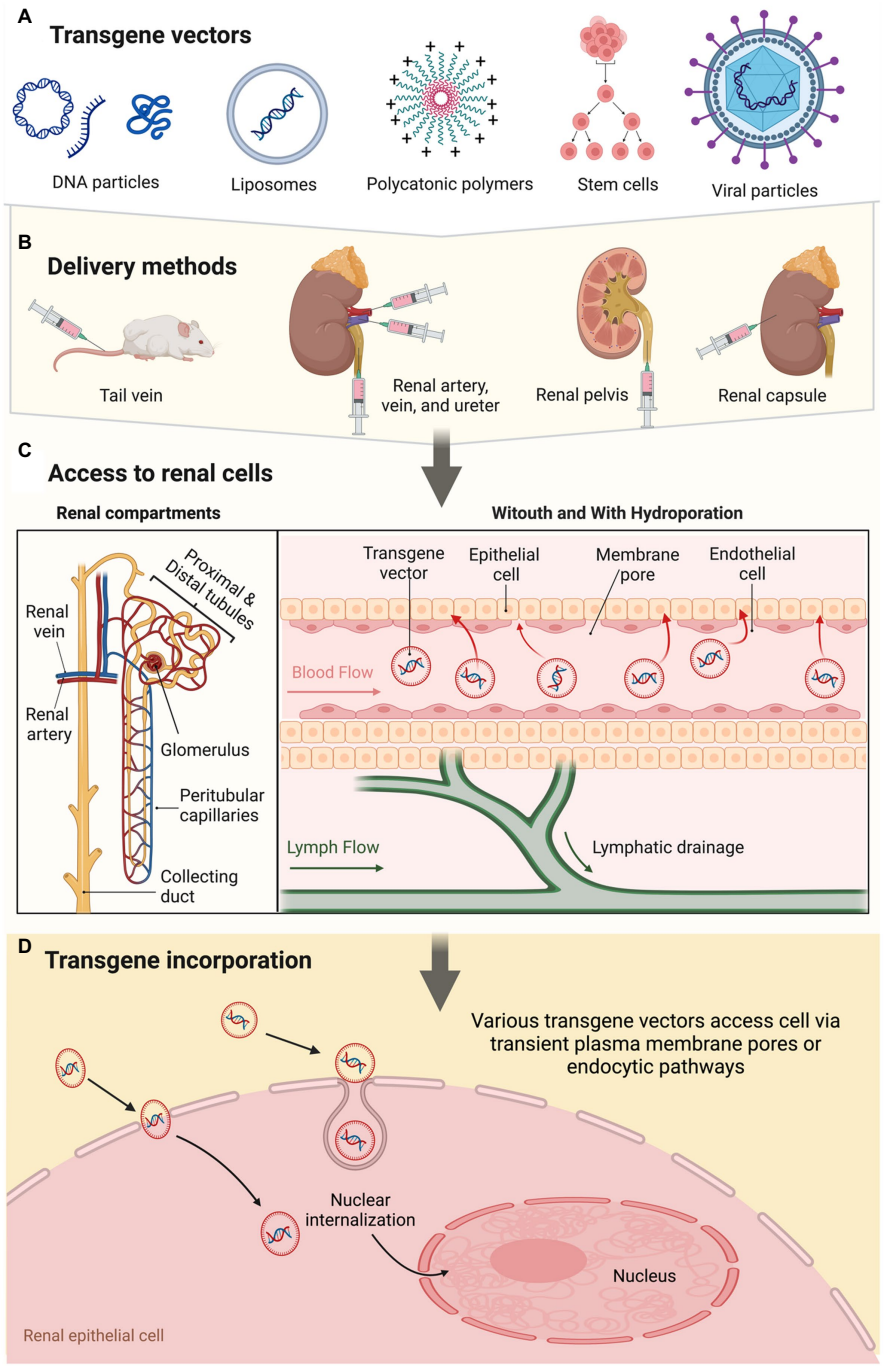
A schematic overview of the renal gene- and cell-based approaches highlights vectorization, delivery mode, and pathways supporting transgene incorporation and expression.

The gene of interest is infused either systemically or directly into the kidney. Apart from the artery, vein, and ureter, direct infusions into the renal capsule and parenchyma using micro-needles (161, 190) or blunt-tip needles (157, 198) have also been proposed, along with indirect tail vein (191, 196, 199) and peritoneum (200, 201) gene delivery schemes. As indicated before, the success of these methods varies per the anatomical location of the targeted cells and the types of vectors used to support gene expression. These vectors include PRC-amplified DNA fragments; plasmid DNA; liposomes; polycations; viral vectors; and stem cells (130). If transformed cells act as gene vectors to promote transgene expression, they may be engineered with various anchoring or binding proteins/peptides to assist their integration into the tissue of interest (202). This process mimics endogenous viral capsid components, which mediate receptor binding and support entry into mammalian cells. As observed in some injured kidney animal models, local healing/regeneration factors facilitate the incorporation of exogenous renal cells delivered intravenously (55). An outline of transgene vector incorporation into the renal epithelium is presented in Figure 1.

Apart from achieving successful genetic modifications, we must also focus on exogenous transgene delivery and expression effects. Such considerations relate to the levels of cellular toxicity and injury that may occur during and after the transfer process. Endo- and exonucleases efficiently degrade DNA fragments (203, 204). However, an overload of exogenous DNA fragmentation may stimulate Ca^2+^ endonuclease activity, degrade endogenous DNA, and mediate cell death (205). Similarly, plasmid DNA, prepared from bacteria, may induce unmethylated CpG motif toxicity that can trigger lower respiratory tract inflammatory responses (206). Oligonucleotide therapies have also been shown to stimulate immune system responses and induce hepatotoxicity and nephrotoxicity (207). Virus-induced toxic and immunogenic responses from high titers, protein overexpression, and capsid protein infections are also topics of significant concern (208). Long-term mutagenesis may also be an issue. Reports have shown such events using recombinant adenovirus systems (209, 210). Specifically, slow-transforming insertional mutagenesis may arise from retroviruses that incorporate into an organism’s genome (211), and *in vivo* stem cell quiescence can tamper with DNA repair mechanisms to further support mutagenesis (212).

## 5. Conclusion

There is a dire need to improve the clinical management of acute and chronic renal diseases. Preliminary outcomes in experimental models with kidney dysfunction managed by gene-based and cell-based approaches are promising. Recent findings echo the traditional need to address several challenges before these therapies become viable clinical options. Existing techniques provide a wide range of success rates and, in some instances, also induce harmful side effects. Thus, further research is needed to develop methods to induce transient or permanent modifications with minimal physiological interference or damage as we aim to improve the treatment of acute and chronic kidney diseases.

## Author contributions

The author confirms being the sole contributor of this work and has approved it for publication.

## Funding

This study was supported in part by the Khalifa University’s College of Medicine and Health Sciences and Grant Number: FSU-2020-25 and funding from RC2-2018-022 (HEIC) awarded to PC.

## Conflict of interest

The author declares that the research was conducted in the absence of any commercial or financial relationships that could be construed as a potential conflict of interest.

## Publisher’s note

All claims expressed in this article are solely those of the authors and do not necessarily represent those of their affiliated organizations, or those of the publisher, the editors and the reviewers. Any product that may be evaluated in this article, or claim that may be made by its manufacturer, is not guaranteed or endorsed by the publisher.
